# Multi-cycle chemotherapy with the glycolipid-like polymeric micelles evade cancer stem cell enrichment in breast cancer therapy

**DOI:** 10.18632/oncotarget.12159

**Published:** 2016-09-21

**Authors:** Tingting Meng, Jingwen Liu, Lijuan Wen, Ming Yuan, Bolin Cheng, Yingwen Hu, Yun Zhu, Xuan Liu, Hong Yuan, Fuqiang Hu

**Affiliations:** ^1^ Institute of Pharmaceutics, College of Pharmaceutical Science, Zhejiang University, Hangzhou 310058, China

**Keywords:** multi-cycle chemotherapy, tumor repopulation, drug delivery, cancer stem cell, microenvironment

## Abstract

Multi-cycle chemotherapy is commonly used in the clinic, while the phenomena of enrichment of cancer stem cells (CSCs) and enhanced multi-drug resistance (MDR) are commonly involved. This research was designed for evaluating this successive administration. Chitosan oligosaccharide-g-stearic acid (CSOSA) polymer was used as the drug delivery system (DDS) to perform tri-cycle chemotherapy on a new tumor model induced by mammosphere cells. *In vitro*, on CSCs enriched mammospheres model, the doxorubicin-loaded CSOSA (CSOSA/DOX) displayed an improved growth inhibition effect measured by acid phosphatase assay (APH). While *in vivo*, the CSOSA/DOX micelles blocked tumor progression and led to a marked decrease of CSCs proportion as well as MDR capacity. What's more, the CSOSA/DOX helped decay the microenvironment and attenuate systemic side effects. We concluded that the CSOSA polymer could be a potential DDS for long-term multi-cycle chemotherapy in antitumor research.

## INTRODUCTION

Chemotherapeutic treatments are commonly used for cancer therapy. However, only specific subgroups of patients are likely to be cured. Dose escalation is not sufficient to result in an improved outcome because the chemotherapy doses are limited by bone marrow suppression and other toxicities [1, 2]. The higher the drug dose is, the greater the cytotoxic effect is. An alternative method for increasing dose intensity would be to use multiple cycles' administration with reasonably short time intervals for recovery of normal tissue. Common used chemotherapies in clinical regimen are multi-cycle and repetitive. However, multiple stimulations of drugs would lead to the enrichment of cancer stem cells (CSCs) [3]. While, the CSCs can drive tumor growth and are responsible for further cancer progression, recurrence and metastasis [4]. Besides, their multi-drug resistance (MDR) capacity [5, 6] would significantly decrease the sensitivity of tumor cells to plenty chemicals and result in treatment failure. Taking strategies to reduce CSCs enrichment would be of vital help in cancer treatment.

Drug delivery system (DDS) has advantages in delivering therapeutic agents and passively concentrating agents within the tumor because of the enhanced permeability and retention (EPR) effect. Targeting strategies have been designed to remove CSCs in tumor [7, 8]. In this research, the glycolipid-like stearic acid-g-chitosan oligosaccharide (CSOSA) polymeric micelles which presented excellent drug accumulation in drug resistant cells [9, 10] were used as a DDS. We aimed to remove both bulk tumor cells and drug resistant CSCs in prevention of their mutual transformation. Besides, three cycles' repetitive chemotherapy with the drug loaded CSOSA micelles was designed to simulate the clinical regimen to detect its antitumor effect and to dig the changing rules of tumor pathology, CSCs proportion as well as MDR capability and the surrounding microenvironment when treated by free drugs and drug loaded DDS.

## RESULTS AND DISCUSSION

### Preparation and characterization of CSOSA/DOX

**Figure 1:**
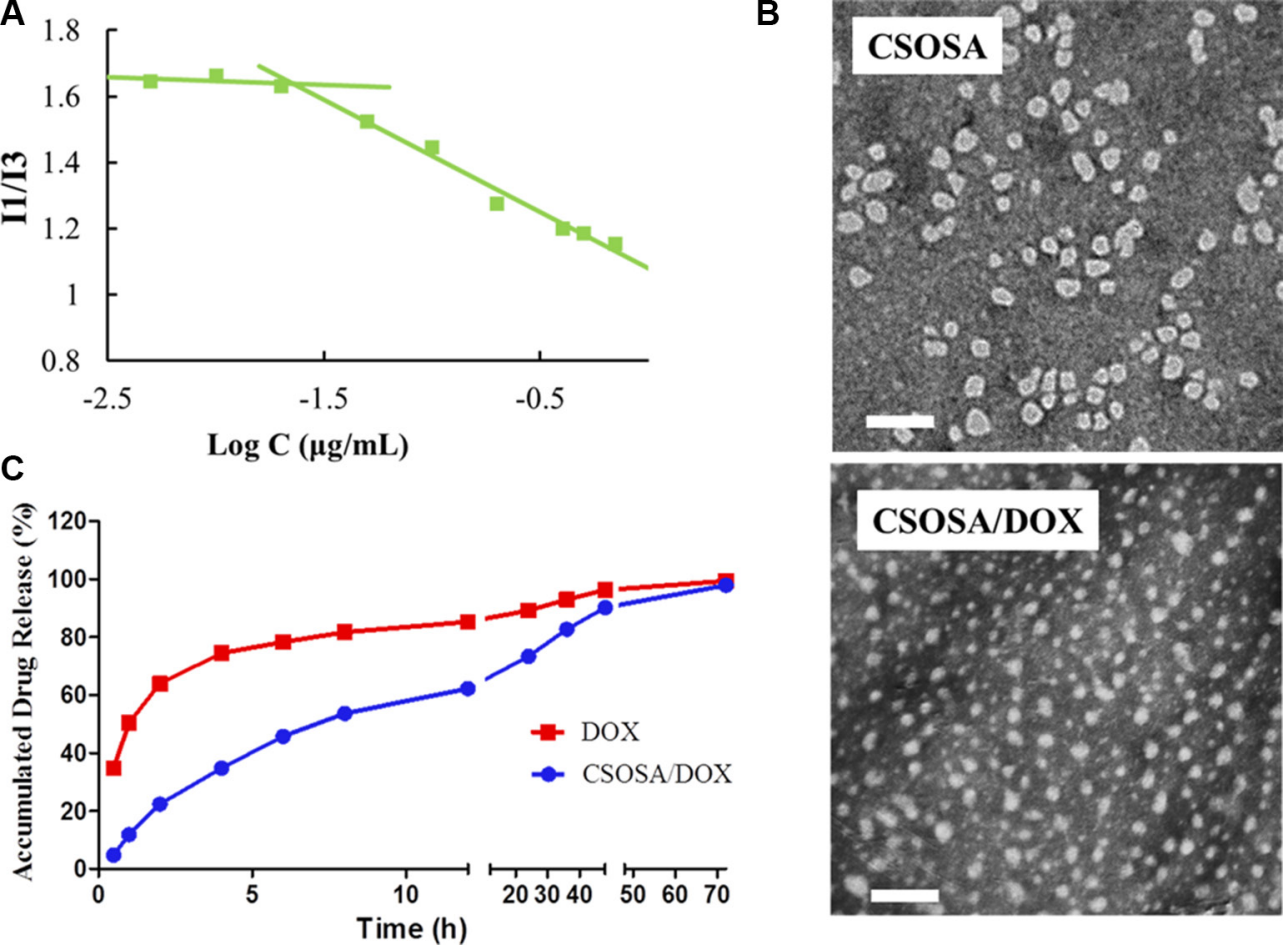
Characteristics of CSOSA and CSOSA/DOX micelles (**A**) The I1/I3 ratio of fluorescence intensity of pyrene against logarithm concentrations of CSOSA. (**B**) TEM images of the micelles. Scale bar, 200 nm. (**C**) *In vitro* DOX release profile of CSOSA/DOX micelles and DOX in PBS (*n* = 3).

The grafted polymer CSOSA self-assembled into micelles with hydrophilic shell and hydrophobic core in aqueous medium. The critical micelle concentration (CMC) value was 22.39 μg/mL calculated according to Figure 1A. Its chemical structure was confirmed by ^1^H NMR (Supplementary Figure S1). After DOX was encapsulated, the particle size of micelles decreased from 53.8 ± 3.2 nm to 34.6 ± 12.7 nm because of the interaction between hydrophobic DOX and micelles' cores. Their size distribution was showed in Supplementary Figure S2. Zeta-potential values for CSOSA and CSOSA/DOX were 21.2 ± 0.2 mV and 22.0 ± 0.7 mV, respectively. Photos taken by transmission electronic microscopy (TEM) (Figure 1B) showed the shape of the micelles with uniform size approximating what was measured above. DOX encapsulated in the micelles also showed a delayed release rate (Figure 1C) indicating its potential as a sustained release DDS.

### Enrichment of CSCs in mammospheres

**Figure 2:**
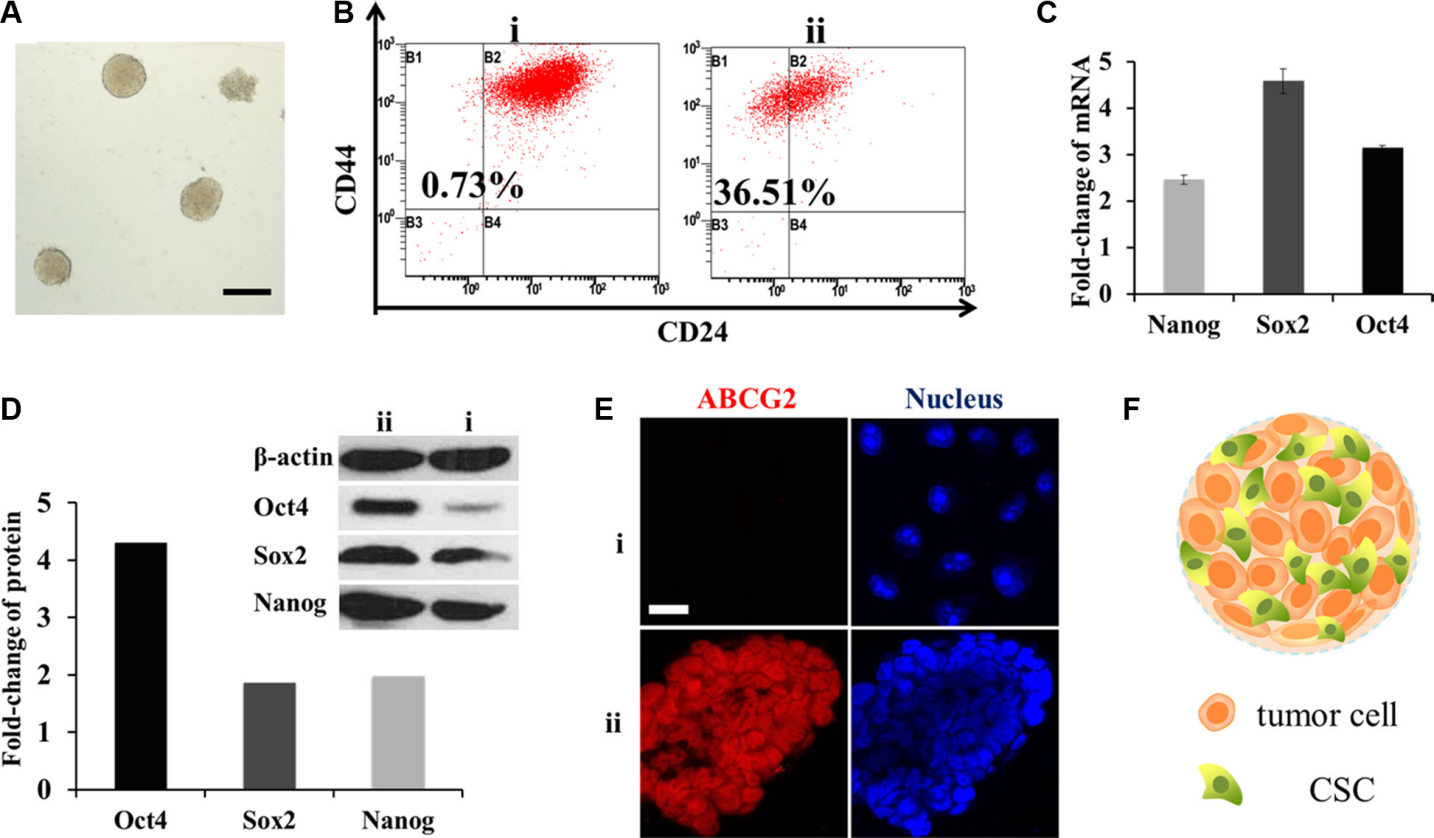
Enrichment of CSCs in mammospheres (**A**) Typical photograph of the mammospheres. Scale bar, 200 μm. i for MCF-7 cells and ii for mammospheres. (**B**) CD44+/CD24− cells measured by FCM. (**C**) mRNA fold change of OCT4, SOX2 and Nanog genes after SFM culture (*n* = 3). (**D**) Fold change of the three proteins. Above: western-blot analysis; below: semi-quantitative analysis by Image J software. (**E**) CLSM for immunostain of ABCG2 (red). Scale bar, 20 μm. (**F**) Schematic diagram of the mammospheres.

The serum-free suspension culture, which attempts to avoid differentiation stimulation by decreasing secretion of differentiation factors and cell adherence, is widely used to obtain CSCs [11]. As shown in Figure 2A, a single MCF-7 cell formed a spheroid structure and became more compact with a well-defined circular shape at the tenth day. We named this structure mammosphere as advised [12]. Following detachment, the cells could reform mammospheres again indicating their ability to self-renew.

To verify the stemness of mammospheres, flow cytometry (FCM) was performed to detect breast CSC surface maker CD44^+^/CD24^−^. The ratio of CD44^+^/CD24^−^ cells in mammospheres was as high as 36.51%, while in MCF-7 cells it was only 0.73% (Figure 2B). OCT4, Nanog and SOX2 genes which were in charge of cell division and differentiation in stem cells [13] were also tested by RT-PCR and western-blot. The mRNA levels were all multiplied, of which SOX2 increased to almost five-fold; while three-fold, two-fold was for OCT4 and Nanog (Figure 2C). Similar to that, from a semi-quantitative calculation of optical density, protein OCT4 was distinctive with three times higher. Meanwhile SOX2 and Nanog were both doubled (Figure 2D). Overall, they were inclined to possess the characters of stem cells.

Another important character of breast CSCs is drug resistance capacity associated with high levels of ABCG2 [14] which defend cells by decreasing cellular accumulation of cytotoxic agents. To observe the expression, mammospheres were frozen sectioned. And then enhanced ABCG2 expression was observed by a confocal laser scan microscopy (CLSM) through the fluorescence of ABCG2 antibody (Figure 2E). These three indexes confirmed that the cells in mammospheres had gained the characteristics of CSCs after serum free medium (SFM) conditioned cultivation. The structure of mammosphere was portrayed in Figure 2F.

### Deeper and more penetration of DOX delivered by CSOSA in mammospheres

**Figure 3:**
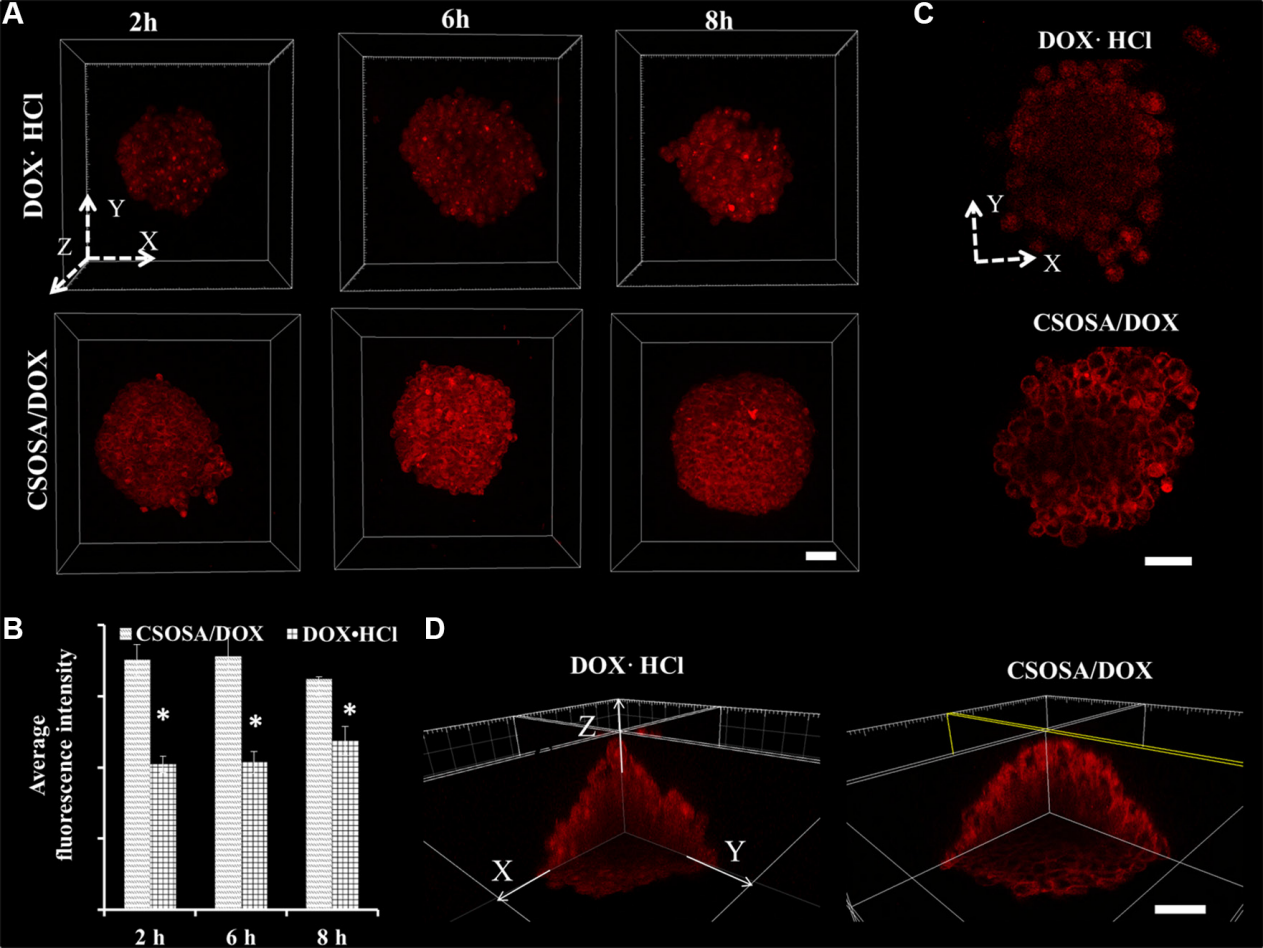
Enhanced uptake of DOX delivered by CSOSA micelles (**A**) Z-stack images of DOX uptake in mammospheres. The arrows marked with X, Y and Z indicated three directions and the images were piled up by XY plane in the Z direction. (**B**) Semi-quantitative analysis of fluorescence intensity in A images calculated by MetaMorph software (*p < 0.05). (**C**) Sheets of XY planes (Z coordinate: 60 μm). Thickness of the planes was 4.8 μm approximating the diameter of a cell. (**D**) Internal cross-sectional view of XY, YZ and XZ planes. Scale bar, 50 μm.

Cellular uptake and penetration experiment of doxorubicin hydrochloride (DOX·HCL) and micelle-loaded DOX were performed on mammospheres. As a 3D structure, resembling tumorospheres, the mammpspheres can reflect nanoparticles penetration besides cellular uptake capability. After incubated with formulations for different time intervals, mammospheres were scanned layer by layer. The 3D images (Figure 3A) were reconstructed by piling up layers at different depths with the Imaris software. Fluorescence intensity in CSOSA/DOX group were all higher than that of DOX·HCl at time intervals 2 h, 4 h and 8 h, which were in accordance with the semi-quantitative results (Figure 3B) (*p < 0.05). Micelle-loaded DOX was internalized slightly after 2 h and with incubation prolonging, internalized DOX started to spread among mammosphere cells. As shown in the XY plane graph (Figure 3C), DOX·HCl showed limited penetration to the outer few cell layers with dispersive diffusion. On the contrary, micelle-loaded DOX efficiently penetrated to the core and mostly distributed in the channel between cells. DOX·HCl's diffusion, which mainly depends on concentration gradient, may be restricted by physical barriers of cell–cell and cell–matrix interactions. Furthermore, rapid uptake, ardent DNA binding capacity and sequestration in acidic endosomes also restricted its penetration to the periphery layers [15]. While, the relatively slow cellular internalization of CSOSA contributed to a decreased cellular consumption of micelle-loaded DOX. Furthermore, lipophilic SA on the surface (so-called minor core) [10] and the positive charge enabled the micelle strongly interact with cell membrane, which could drag micelles to the membrane of more inner cells. In consideration that CSCs were usually harbored in the center of a tumor [16], taking the DDS as a strategy would be accessible for drugs to reach them. For direct observation of the internal drug distribution, 3D cross-sectional images (Figure 3D) and videos (Supplementary materials) were showed. The fluorescence intensity weakened from the outer side to the inside core.

### Enhanced mammosphere suppression in CSOSA/DOX group

**Figure 4:**
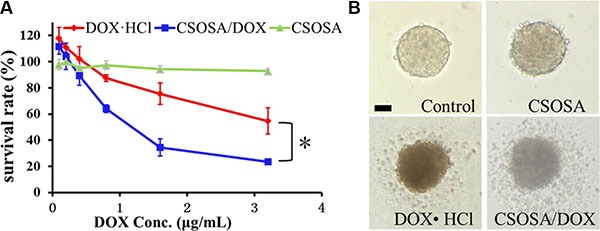
Cytotoxicity of CSOSA/DOX against mammospheres (**A**) The survival rates of the mammospheres measured by APH assay. *p < 0.05, *n* = 3. (**B**) Typical light photographs of mammospheres treated with formulations (CSOSA, DOX·HCl, CSOSA/DOX). The equivalent dose of DOX was 1.5 μg/mL. Scale bar, 50 μm.

Since the regular MTT assay has a limitation of passing through cells in periphery layers and reacting with internal ones (Supplementary Figure S3), an acid phosphatase (APH) assay was adopted for detection of all mammosphere cells with the addition of Triton-X-100. Blank CSOSA at relevant concentrations showed barely cytotoxicity (Figure 4A), which indicated its safety. DOX encapsulated in CSOSA showed enhanced suppression effect with a decrease of IC_50_ value from 2.64 μg/mL to 1.07 μg/mL (*p < 0.05). The reason for stronger CSOSA/DOX suppression effect may due to its increased intracellular concentration. And also CSOSA hold the potential to gather into cell nucleus [17] where DOX mainly works. It could also avoid the efflux by ABC transporters which highly expressed in breast CSCs. In contrast, DOX·HCl could be recognized and excluded. In the curve, it's interesting to see that the survival rate of the first two points rose above 100%, which may be attributed to the released prostaglandin E2. It was produced by chemotherapy damaged cells and could recruit CSCs into cell division [18].

For direct evaluation, spheroid visualization was introduced. Mammospheres without any drug treatment were bright with a distinct border and cells tightly bonded with each other (Figure 4B). While, the CSOSA-treated mammospheres resembled the control except for a reduced refractivity. Cells began to shrink and fall apart in DOX·HCl and CSOSA/DOX treated groups; more shed and broke up in the latter group, indicating an enhanced suppression effect of CSOSA/DOX on breast CSCs.

### Obvious distinction between MCF-7 and MCF-7 CSCs induced orthotopic xenograft tumors

**Figure 5:**
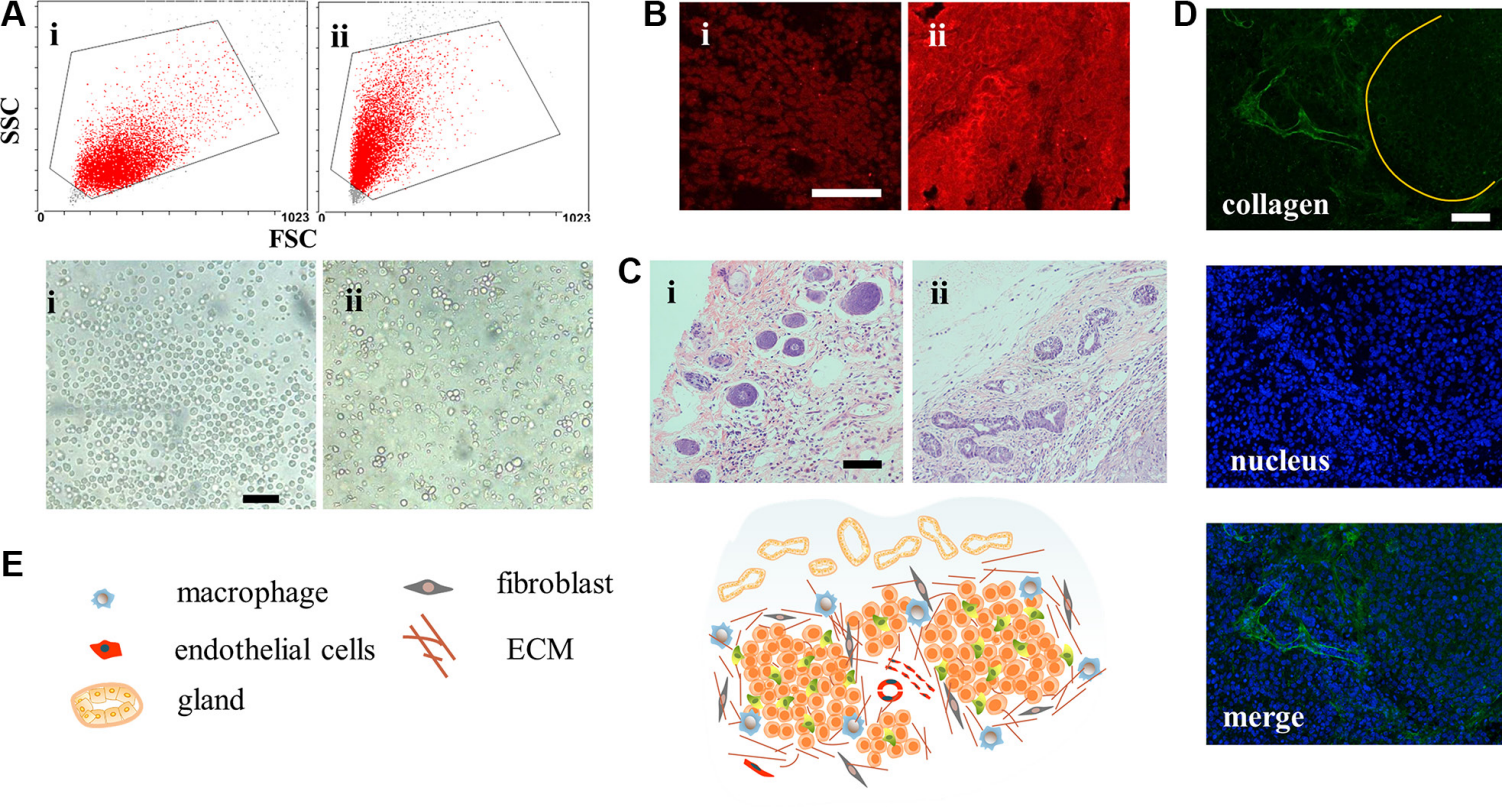
Structure of the CSCs induced orthotopic xenograft tumor model (**A**) Conventional FCM FSC/SSC analysis and light photographs of the tumor cells. Scale bar, 50 μm. i for MCF-7 cells and ii for mammospheres. (**B**) CLSM for immunostain of ABCG2 (red). Scale bar, 100 μm. (**C**) H&E stain images of the mammary tissue on the peripheral region of the tumors. Scale bar, 200 μm. (**D**) The clusters of tumors were surrounded and separated by collagen. (**E**) Structure and cell components scheme of CSCs-induced orthotopic tumor.

CSCs have strong tumorigenicity with as little as 100 cells [19]. However, few studies had reported the difference between tumors induced by CSCs and normal cancer cell lines. In this study, mammosphere cells and MCF-7 cells were inoculated under the mammary gland, respectively. Volumes of MCF-7 tumor grew up to 200 mm^3^ approximately 10 days after injection of MCF-7 cells and the MCF-7 CSCs tumors retarded for a few days. Tumors reaching 500 mm^3^ were applied to explore their distinctions. However, special higher expression of CD44^+^/CD24^−^ cells was not distinguished from CSCs tumor cells, similar to what was reported [20]. Interestingly, cells digested off from the tumors displayed different morphology (Figure 5A). According to the conventional FCM FSC/SSC analysis, where values for X mean and Y mean respectively represented cell diameter and internal granularity, the MCF-7 tumor cells were bigger in size and with fewer organelles. As shown in the microscope photos, they were perfectly round and uniform. On the contrary, morphology of CSCs tumor cells was diverse with round, polygonal or rectangular shapes, which suggested that the cells differed within the CSCs tumor model. The stem-like cells (CD44^+^/CD24^−^ cells), accounting for 36.51% in mammospheres, may attribute to the diverse cell types. Just as that in the serial tumorigenicity assay, CSCs could generate new tumors containing additional CSCs as well as phenotypically diverse mixed populations of non-CSCs, which made them resemble the primary tumors [19, 21].

To figure out whether the drug resistance capacity was still maintained after inoculation, expression of ABCG2 protein was explored. From Figure 5B, we found that MCF-7 CSCs tumors showed brighter red fluorescence meaning more ABCG2 proteins in the early state, which would equip the tumor with feature of drug resistance. It's worth noting that many cancers in the clinic had intrinsic resistance, not acquired resistance capacity. The structure of surrounding breast tissue was also changed, showing more ductal elements in the specific mammary gland conformation (Figure 5C). The increased ductal branching is usually related to mammary tissue remodeling. Previous research had demonstrated that between non-CSCs and CSCs, only the CSC fraction remodeled the stroma environment [22]. This provided additional evidence for the stemness property of mammospheres. With more ductal elements, the basement membrane would block access of DDS [23]. However, the effect of remodeling in tumor still remains to be estimated. The CSC tumor structure was also special with cell clusters surrounded by collagen (Figure 5D) and the whole structure was portrayed in Figure 5E. These properties collectively demonstrated that the MCF-7 CSC-induced orthotopic xenograft model, different from MCF-7, recapitulated the complexity of primary tumors, with tumor heterogeneity, drug resistance ability and ductal elements.

As to *in vivo* preclinical research, a proper tumor model is always of vital importance and also a major stumbling block. The preclinical knowledge has been acquired principally from cell line-derived xenografts or patient-derived xenografts (PDXs). However, the cell line-derived xenografts are poorly predictive due to genetic drift and losses of tumor heterogeneity [24] and PDXs are cumbersome and costly [25]. The CSC orthotopic xenograft model could meet the above requirements. Additionally, orthotopic organ environment could avoid the formation of skin ulcerations [26], which is benefit for long-term experiment. Above all, this provided a useful platform of tumor model for studying DDS.

### Blocking tumor repopulation by CSOSA/DOX

**Figure 6:**
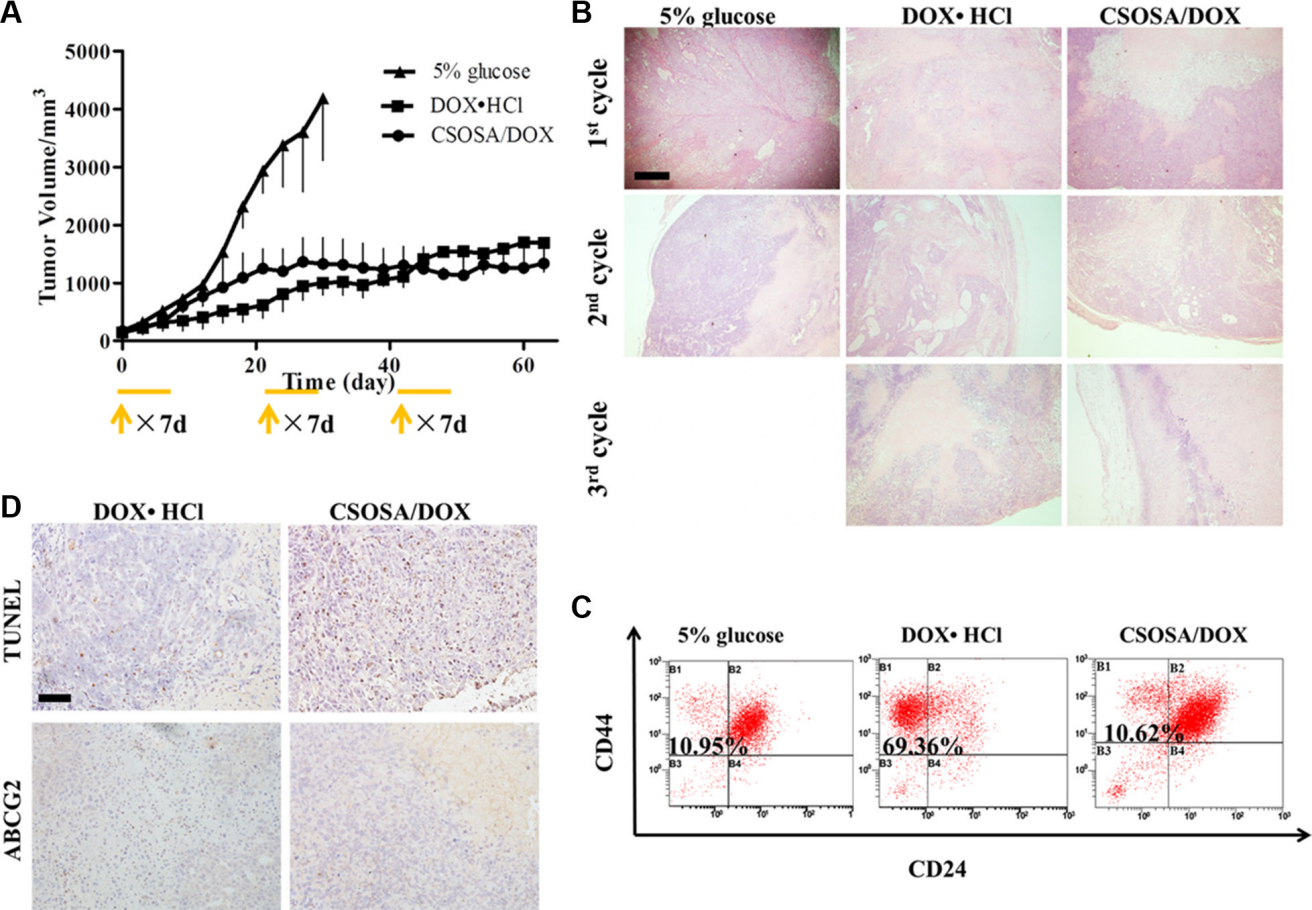
Tumor repopulation blocking effect of CSOSA/DOX (**A**) Tumor growth curve after injection of the formulations (*n* = 4). The start of each treatment cycle was noted with arrows and each administration cycle last for 7 d. (**B**) H&E analysis of tumors at the end of each cycle. The pink area represented necrosis. Scale bar, 500 μm. (**C**) FCM histograms of the CD44^+^/CD24^−^ cells in B1 section. (**D**) TUNEL was for apoptosis and ABCG2 stain for drug resistant capacity. Scale bar, 200 μm.

Multi-cycle repetitive administration was designed to simulate clinical regimen. Tumor naturally expanded exponentially from beginning to end in the glucose group (Figure 6A). Following the guidelines of the Institutional Animal Care and Use Committee, mice were sacrificed when their tumor volumes reached 4000 mm^3^. Significant enhanced antitumor activity was observed in the DOX·HCl and CSOSA/DOX groups compared to the glucose group (**p < 0.01). The whole growth of the two groups showed no significant difference (*p* > 0.05), whereas when analysed separately by cycles it tended the other way (1st, 2nd, 3rd cycle; *p* > 0.05, ***p < 0.001, **p < 0.01). Although tumors treated with DOX·HCl was small and grow rather slowly, it continued expanding all the time and showed an accelerated repopulation at the start of the successive cycle after the intervals (Supplementary Figure S4). The spaced treatments were intended for recovery of normal tissue, however tumors also recovered and proliferated. The CSOSA/DOX showed a weaker or retardant antitumor effect in the 1st cycle (the first 21 d), which may be attributed to the slower uptake and release rate of DOX. But it stopped growing since the second cycle. Compared to DOX·HCl, CSOSA/DOX showed superior antitumor activity since 2nd cycle, maintaining a stable size without repopulation. The tumor suppression effect of CSOSA/DOX could compete with that of DOX·HCl and be more effective for the perspective of long-term chemotherapy.

To see the internal growth status, tumors at the end of each cycle were stained with hematoxylin and eosin (H&E). Normal tumor cells had large blue nucleus, whereas, the chromatin of necrotic cells became absent with only cytoplasm stained red. Degree of necrosis, to some extent, represented therapeutic efficiency. The negative group showed the largest and thickest living area (Figure 6B) and its necrosis was probably due to a deficit supply caused by rapid growth. Large necrosis areas were observed in the other two groups, and larger in CSOSA/DOX group, where necrosis level became higher with treatment time prolonging. However, in the DOX·HCl group there were always blue areas inside the tumor, revealing vigorous regrowth ability.

### Better prognosis in CSOSA/DOX- treated tumors

Since CSCs were responsible for cancer progression, recurrence and metastasis, the treated tumors were extracted to detect its variation of the CD44^+^/CD24^−^ cell percentage. As indicated in Figure 6C, the CSCs proportion of DOX·HCl group was remarkably increased with a proportion of 69.36%, seven-fold of that in glucose and CSOSA/DOX groups (10.95% and 10.62%). This suggested the CSOSA/DOX would not lead to CSCs enrichment and killed non-CSCs and CSCs indiscriminately, which was essential in tumor therapy [27], because the non-CSCs could transform into CSCs and further sustain tumor growth [28, 29]. Actually in the CSOSA/DOX group, the tumor volume was much smaller, thus the real number of CSCs was far less.

After pulsed stimulation for three cycles, it was quite understandable for tumors to express increased ABCG2 protein (Figure 6D). ABCG2 was firstly found in the second cycle (Supplementary Figure S5D), implying that the acquiring process needed time. On the contrary, no ABCG2 was found in the other two groups meaning no acquired resistance occurred. Pictures of apoptosis were taken at the non-necrotic area, in case being affected by the necrotic cells. Apoptosis randomly occurred in the glucose group; more apoptotic areas were obviously observed in CSOSA/DOX group compared to DOX·HCl group.

The CSOSA/DOX group exhibited smallest living area but strongest apoptosis as well as weakest drug resistance, suggesting a better therapeutic effect. As for DOX·HCl, many factors related to its ever growing. Initially, the CSCs tumor model at the early stage was drug-resistant and DOX·HCl can be extruded. Subsequently, tumors got stronger resistance capacity with DOX·HCl pulse simulation and become more difficult to kill. Another reason for its repopulation was the remaining CD44^+^/CD24^−^ cells which reserved the ability to proliferate, differentiate and ultimately facilitated tumor growth.

### Microenvironment damage caused by CSOSA/DOX

**Figure 7:**
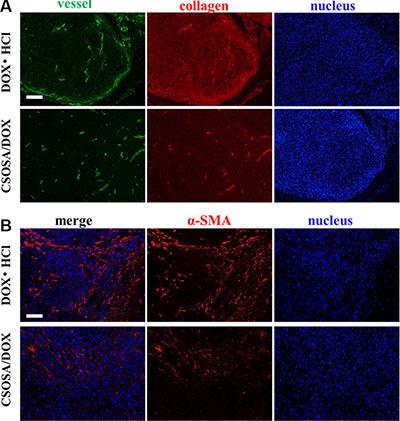
Damage to the microenvironment caused by CSOSA/DOX (**A**) Representative images from immunofluorescence stain of tumor vessels (green) and collagen (red). (**B**) Immunofluorescence images of CAF (α-SMA, red). The CAFs in the DOX·HCl group was more highly and separated the tumor cells into clusters. Scale bar, 200 μm.

CSC niche was further explored to explain the suppression effect. Except for supporting tumor cells, microenvironment also acted as barriers hindering delivery of nanoparticles. The dense collagen matrix reduced vascular perfusion through elevated interstitial fluid pressure and compressing tumor vessels [30–32], and thus resulted in suboptimal outcomes [33]. As shown in Figure 7, after treated by CSOSA/DOX, vessels became bigger and more. This would enlarge the vascular perfusion for more drug distribution. Generally, free chemicals are unable to penetrate more than 40–50 μm from vessels because of extracellular matrix, let alone the distance between tumor cells and vessels was often more than 100 μm [34]. However, CSOSA/DOX resulted in lower collagen levels and made it easy to penetrate and to reach tumor cells. In addition, the particle size of the micelle was appropriate for tumor penetration, as previous research reported only micelles of 30 nm could penetrate in poorly permeable pancreatic tumors among different sizes [35]. While, in DOX·HCl group, tumor vessels were squeezed by dense collagen. The situation was the same at the end of each chemotherapy cycle (Supplementary Figure S5A–S5C) and the collagen grew more and more tense. Furthermore, the destroyed microenvironment, niche for CSCs, could no longer modulate the transformation of CSCs to non-CSCs [36]. Tumor cells to some extent were harbored and protected by secreted collagen. After treated by CSOSA/DOX, shield for tumor cell was lost. Thereby the chemotherapy was potentiated.

To figure out the leading cause of decreased collagen level, cancer-associated fibroblast (CAF), secreting collagen in tumors [37], was traced (Figure 7B). Interestingly, CAFs in CSOSA/DOX showed dispersed distribution and was of low content. It implied that the CAF was an acting target of CSOSA/DOX. On the contrary, tumors in DOX·HCl group were abundant of CAFs and clusters of tumor cells were separated by them.

### Reduced systemic toxicity of DOX by CSOSA micelles

**Figure 8:**
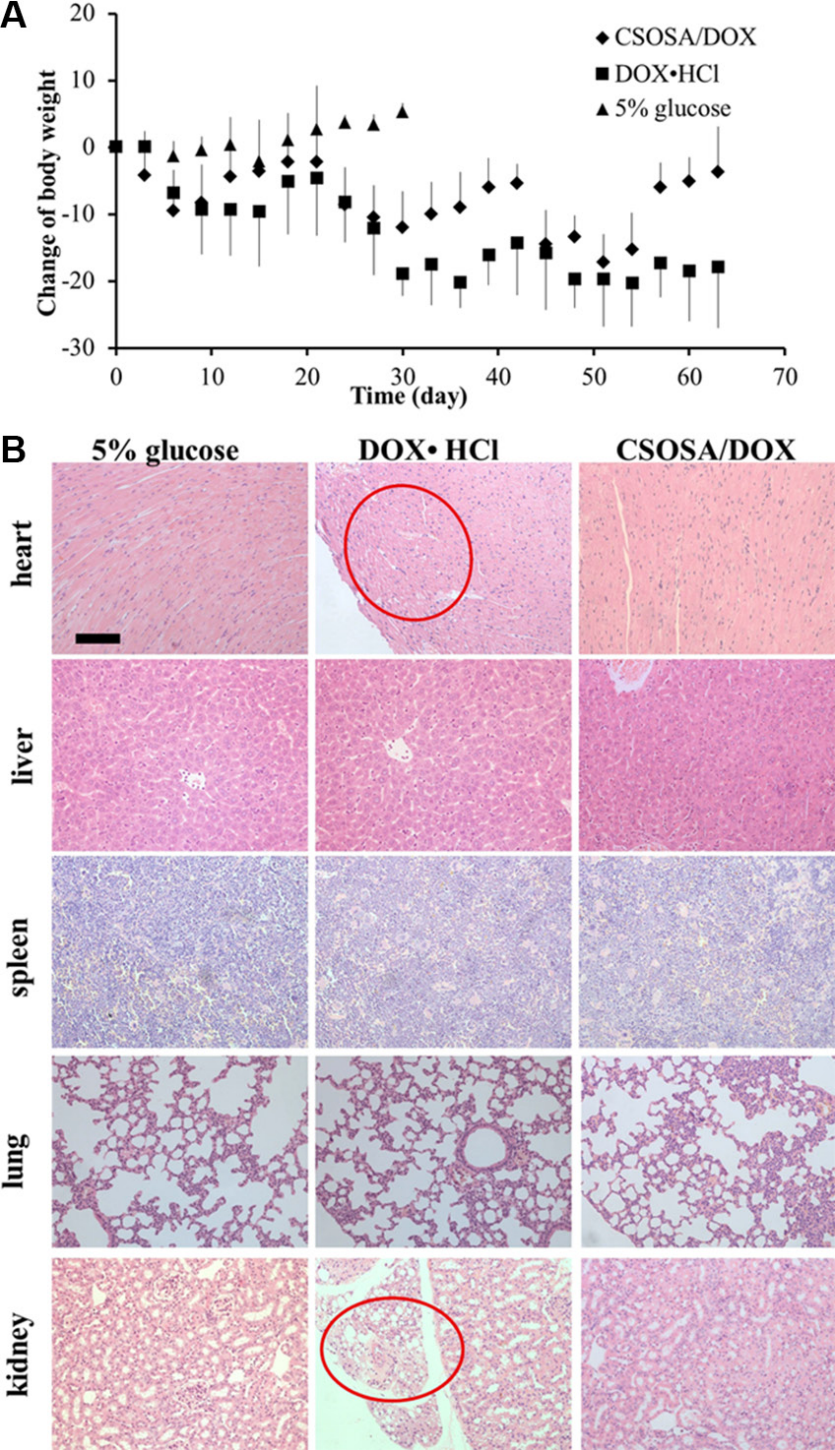
Systemic toxicity caused by DOX (**A**) Changes of body weights with repetitive administration of three formulations. (**B**) H&E analysis of the organs at the end of the treatment. Myofibrillar loss in hearts and vacuoles in kidneys were indicated by the red cycles.

Besides therapeutic efficacy, safety was another important therapy index. Low cytotoxicity was undoubtedly a huge advantage especially with this long-term administration. The body weight decreased after administration of drugs (Figure 8). Mice in CSOSA/DOX group could always recover during the intervals. While mice in the DOX·HCl group would only regain a bit and this regain get less and less as chemotherapy time extended. To further characterize its toxicity, a histopathological examination of internal organs was applied. Slight extramedullary hematopoiesis was found in all liver and spleen because of long-term burden with tumor. Organs of CSOSA/DOX-treated mice maintained regular cell distribution and normal architecture. However, irregular cell arrangement and myofibrillar loss in hearts and vacuoles in kidneys were serious in the DOX·HCl group (Figure 8B). For the CSOSA micelles alone, the chitosan oligosaccharide was biodegradable, biocompatible and non-toxic material. The reduced systemic toxicity of DOX encapsulated could be credited to the changed distribution by CSOSA micelles.

## MATERIALS AND METHODS

### Materials

Chitosan oligosaccharide (CSO, Mw = 18 kDa, 95% deacetylated degree) was obtained by enzymatic degradation of chitosan (Mw = 450.0 kDa) supplied by Yuhuan Marine Biochemistry Co., Ltd. Stearic acid (SA) was purchased from Aladdin Industrial Inc. DOX was purchased from Hisun Pharmaceutical Co., Ltd. Insulin and immunopure p-nitrophenyl phosphate were purchased from Sigma Chemical Co. EGF and bFGF was purchased from Peprotech. RNAiso Plus, PrimeScriptTM RT reagent Kit and SYBR^®^ Premix Ex TaqTM were purchased from TaKaRa Biotechnology (Dalian) Co., Ltd. SOX2, NANOG and OCT4 antibodies were purchased from ABGENT.

MCF-7 cells were obtained from Cell Resource Center of China Science Academe. Female BALB/c nude mice (6–8 weeks old) were purchased from Shanghai Silaike Laboratory Animal Limited Liability Company. The mice were raised in the specific pathogen-free level animal facility and freely allowed access to food and water.

### Synthesis of CSOSA and preparation of CSOSA/DOX

CSOSA was fabricated via the reaction of the carboxyl group of SA with the amine group of CSO in the presence of 1-ethyl-3-(3-dimethylaminopropyl) carbodiimide (EDC) as reported [10]. Briefly, SA (0.120 g) and EDC (0.430 g) dissolved in 18 mL ethanol were maintained at 60°C to activate the carboxyl group. CSO (0.5 g) dissolved in 36 mL distilled water was pre-heated followed by addition of the activated mixture. After stirred at 60°C overnight, the final reaction mixture was dialyzed against distilled water. Then, the lyophilized product was dispersed in ethanol to remove the unreacted SA by filtration. The CSOSA was received after lyophilized.

DOX-loaded micelles were prepared by dialysis. Briefly, a solution of DMSO containing DOX was dropped in the micelle solution and stirred for 1 h, followed by dialysis against distilled water (Milli-Q, Millipore) overnight. Then, the unencapsulated DOX was removed by centrifugation at 8000 rpm for 10 min (3K30, Sigma Laborzentrifugen).

### Characterization of CSOSA and CSOSA/DOX

The average particle diameter and size distribution of blank micelles and DOX-loaded micelles were measured by a dynamic light scattering (DLS) Zetasizer (3000HS, Malvern Instruments Ltd, UK). The samples were placed on copper grids and stained with 2% (w/v) phosphotungstic acid for viewing by a transmission electron microscopy (TEM, JEM-1230, Japan). The critical micelle concentration (CMC) of the CSOSA was determined by pyrene fluorescence method using a fluorometer (F-2500, Hitachi Co., Japan). Pyrene (6.0 × 10^−7^ mol/L) was pre-dried and CSOSA solutions were added and sonicated together for 30 min. The excitation wavelength was set as 337 nm and the emission wavelength was scanned.

*In vitro* cumulative release of DOX from the micelles was investigated in PBS. First, 1 mL CSOSA/DOX solution was introduced into a dialysis membrane (MWCO = 3.5 kDa, Spectrum Labs, USA) with ends sealed and submerged into 20 mL release medium. The system was stirred at 60 rpm and maintained at 37°C. Samples were withdrawn at predetermined times (1 h, 2 h, 4 h, 6 h, 12 h, 24 h, 36 h and 48 h) from the release medium. The concentrations of released DOX were determined by a fluorometer. As control, dispersion of DOX was conducted under the same condition.

### Formation of mammospheres

A single-cell suspension of MCF-7 cells suspended at a density of 5 × 10^3^ cells/mL in serum free medium (SFM) was inoculated in ultra-low-attachment plates. The SFM was DMEM/F12 medium supplemented with human recombinant epidermal growth factor (20 ng/mL), recombinant basic fibroblast growth factor (20 ng/mL), 5 μg/mL insulin and 2% B27 supplement. The cells were cultured for about 10 d in an incubator at 37 °C in humidified 5 % CO2 atmosphere. The medium was added every 5 d.

### Flow cytometry

Mammospheres were trypsinized into single-cell suspension with Accutase-Enzyme Cell Detachment Medium, and then washed and resuspended in PBS. Single cells were labeled with antibodies specific for human cells: anti-CD24-FITC and anti-CD44-PE (BD PharmingenTM). An unstained, single stain served as the control. Isotype controls were used to exclude non-specific conjunctions. After being incubated with antibodies for 30 min at 4°C in the dark, the unbound antibody was washed. Cells were fixed for analysis (FC500MCL, Beckman Coulter, USA).

For tumor analysis, harvested tumors were minced to form single cell suspension. The tissue lysate was filtered through a 200 mesh sieve prior to stain. Surface antigen CD24 and CD44 were detected as above. The single cell suspension was also observed under a light microscope.

### Real-time polymerase chain reaction

Total RNA was extracted by TRIzol reagent (Gibco BRL, Gaithersburg, MD) according to the manufacturer's protocol. Nucleic acid concentrations were measured at 260 nm (Nanodrop2000 Spectrometer). One microgram of RNA sample was reverse transcribed to cDNA with the PrimeScript^™^ RT Reagent Kit. The cDNA synthesis condition was 37°C 15 min and 85°C 5 s. A master mix for each PCR run was prepared with SYBR^®^ Premix Ex Taq^™^ reagent. Appropriately diluted cDNA was added with the primers (sequences shown in Supplementary Table S1). The PCR condition was 94°C for 3 min to denature the RNA/cDNA hybrid, then 40 cycles of 94°C for 1 min, 45°C for 1 min, and 72°C for 1 min. All samples were amplified in triplicate (StepOneTM, Applied Biosystems, USA). The comparative cycle threshold (CT) (2^−ΔΔCT^) method was used to determine the relative expression.

### Western blot analysis

The MCF-7 cells and mammospheres were mixed with sample loading buffer, destroying the cell membrane with pipette tip, boiled for 15 min and then separated by Tris-Tricine SDS-PAGE. Proteins were electrophoretically transferred to nitrocellulose membranes and blocked with skim milk in PBS containing 0.05% Tween 20 for 1 h. After we washed the membrane, the antibodies of Nanog, OCT4 and SOX2 were added and incubated for 4 h at room temperature. The bound antibodies were detected by horseradish peroxidase-conjugated IgG secondary antibodies. Signals were developed via an enhanced chemiluminescence detection system.

### Uptake of DOX·HCl and CSOSA/DOX in mammospheres

Mammospheres approximately 200 μm in diameter were incubated with DOX·HCl and CSOSA/DOX. The images were collected on a confocal laser scan microscopy (CLSM) (BX61W1-FV1000, Olympus) with a 40× water immersion objective. Laser beam with 546 nm excitation wavelength was used for DOX. Z-stack images were obtained by scanning the mammospheres layer by layer. Each scanning layer was 1.2 μm in thickness, and the total scanning was approximately 100 μm in depth.

### Mammosphere suppression measured by APH assay

The *in vitro* cytotoxicity was evaluated by APH assay with minor modification [38, 39]. Mammospheres grew in 96-well plates with 150 μL medium was incubated with CSOSA, DOX·HCL and CSOSA/DOX for 48 h, followed by centrifugation to spin down and washing with PBS. The supernatant was discarded to a final volume of 100 μL. Then, 100 μL of the assay buffer (0.1 M sodium acetate, 0.1% Triton-X-100, 1 mg/mL immunopure p-nitrophenyl phosphate (Sigma)) was added and incubated for 90 min at 37°C. After supplemention of 10 μL NaOH solution, absorption at 405 nm was measured on a microplate reader (SpectraMax M5, Molecular Devices).

### Tumor progression studies

All of the experiments were performed in compliance with the guidelines established by the Zhejiang University Institutional Animal Care and Use Committee. The mice were treated with estrogen before inoculation. The MCF-7 and mammosphere cells were injected into the mammary fat pad of the 4th nipple. An estradiol supplementation was given every 5 d until the tumor come up.

Tumor-bearing mice were randomly divided into three groups: 5% glucose control group, positive control group (DOX·HCl, 2 mg/kg/d in 0.2 mL) and the CSOSA/DOX group (DOX, 2 mg/kg/d in 0.2 mL). Three chemotherapy cycles were performed and each was 21 d. Drugs were administered intravenously in the first 7 d of each cycle. The tumor size and body weights were recorded every 3 d. The volume was calculated with the formula: (length) × (width)^2^ /2. For the analysis of changes between cycles, two more mice were added in each group and sacrificed at the end of each cycle.

### Histological analysis

Tumor and organ samples were fixed in 4% paraformaldehyde and embedded in paraffin before sectioning at 4 μm thickness. H&E staining was performed as follow: After fixed with ethanol for 20 min, sections were washed and treated with hematoxylin for nuclei staining. Then, after washed, the cytoplasm was counterstained with 1% eosin solution. Stained sections were embedded in glycerin jelly followed by final water washing.

Immunohistochemistry and immunofluorescence were performed as follow: after fixed with cold acetone, cells were blocked with 2% goat serum for 2 h. The primary antibody of ABCG2 (collagen type), α-SMA, CD31) were applied to samples overnight at 4°C. After washing, the secondary antibody was incubated for 1 h. Nuclei were stained with DAPI for 10 min. The samples were protected by glycerol and covered by a cover glass.

### Statistical analysis

All of the data were expressed as means ± standard deviation (SD). Statistical significance was analyzed using Student's *t*-test. *p* value of < 0.05 was considered statistically significant. *p* > 0.05 represents not significant; *p < 0.05; **p < 0.01; ***p < 0.001.

## SUPPLEMENTARY MATERIALS FIGURES, TABLE AND VIDEOS










