# Supplementary material for: Multi-cycle chemotherapy with the glycolipid-like polymeric micelles evade cancer stem cell enrichment in breast cancer therapy
Source: Oncotarget. 2016 Sep 21;7(45):72978–89. doi: 10.18632/oncotarget.12159 (PMC5341957; doi:10.18632/oncotarget.12159)
Supplement: Supplementary file 1 [file oncotarget-07-72978-s001.pdf]

Multi-cycle chemotherapy with the glycolipid-like polymeric micelles evade cancer stem cell enrichment in breast cancer therapy

Supplementary Materials

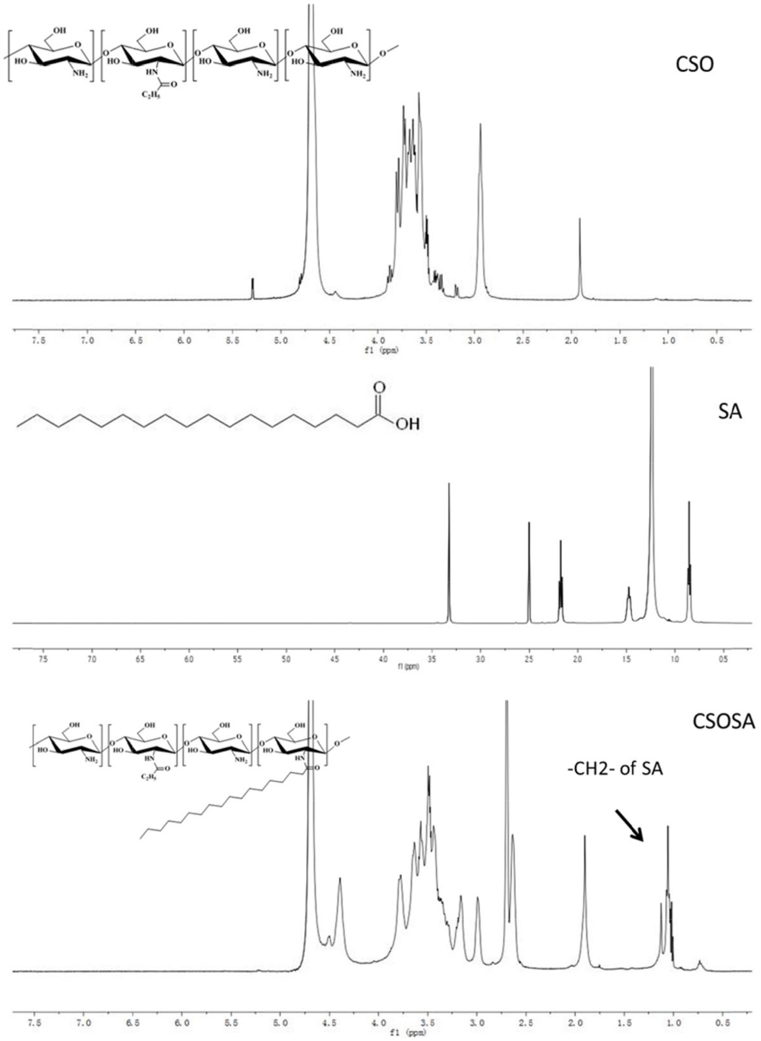

Supplementary Figure S1: Chemical structure verification of CSOSA polymer. ^{1}H NMR spectra of CSO, SA and CSOSA.

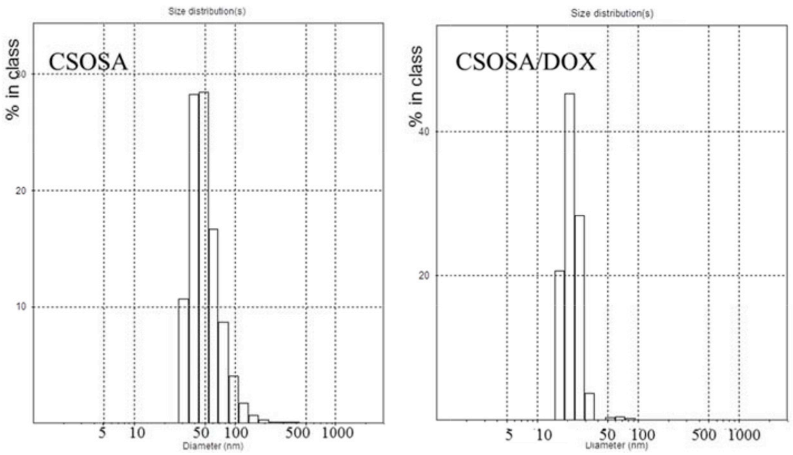

Supplementary Figure S2: Size distribution of CSOSA and CSOSA/DOX micelles.

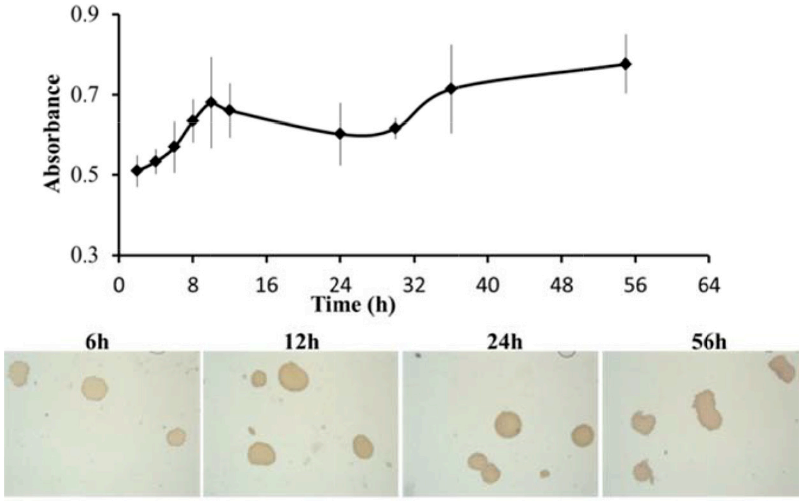

Supplementary Figure S3: MTT assay performed on mammospheres. (**A**) The absorbance of formazan showed irregular variation and changed little with time extended. (**B**) The images of the mammosphere cores at different time intervals. No purple formazan was produced.

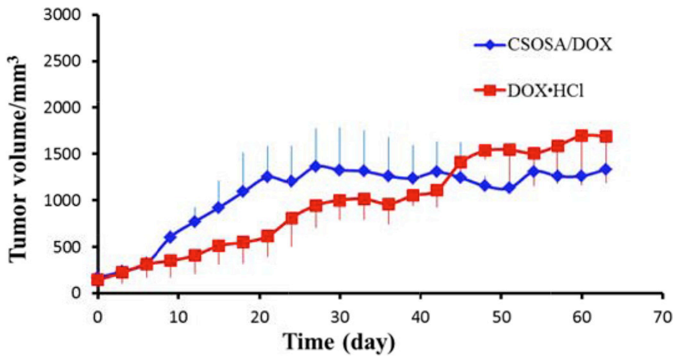

Supplementary Figure S4: Tumor growth curve after injection of the formulations (CSOSA/DOX and DOX•HCl). Glucose group was deleted to highlight the growth changes of the other two groups.

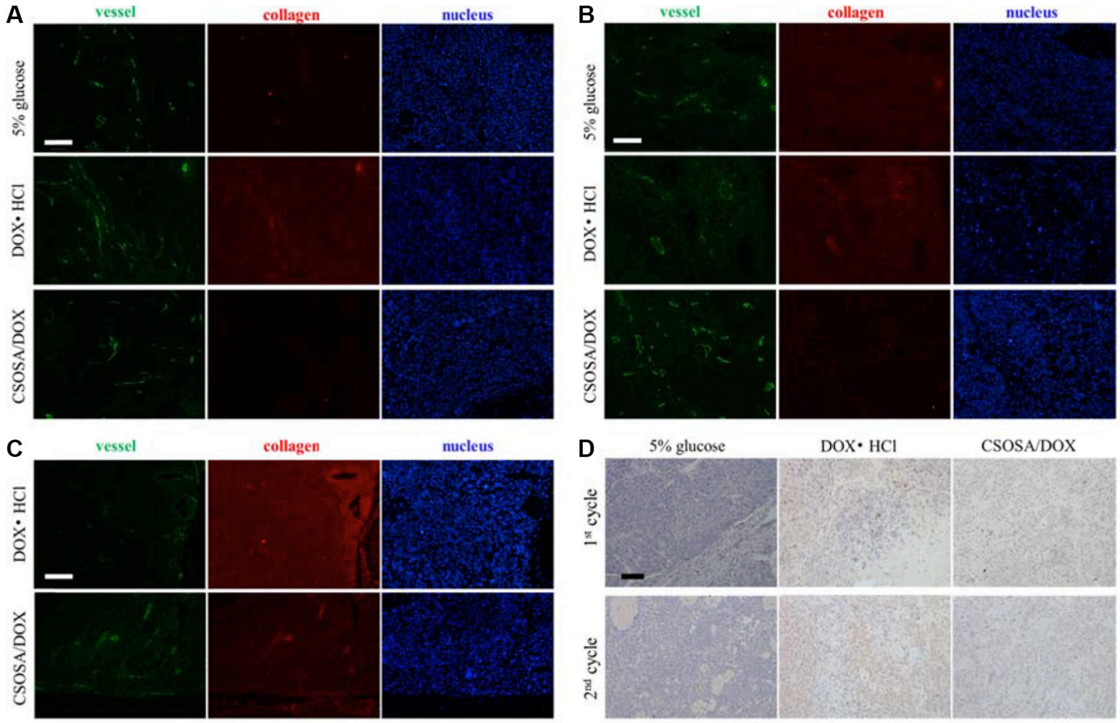

Supplementary Figure S5: Information got during the treatment process. (**A**–**C**) successively represents change of the content of collagen and vessels at the end of the 1st, 2nd and 3rd cycle. The collagen (red) grew more and more intense in the DOX•HCl group and changed little in CSOSA/DOX group. On the contrary, the vessels (green) were bolder. The blue indicates neclues. Scale bar, 500 µm. (**D**) Expression of ABCG2 in the first two administration cycles. The level of ABCG2 was all very weak. A slight expression was seen in the 2nd cycle of DOX•HCl group. Scale bar, 200 µm.

Supplementary Table S1: The sequence of the primers used in the RT-PCR

Primers	Forward primer (5′-3′)	Reverse primer (5′-3′)
GAPDH	GCACCGTCAAGGCTGAGAAC	TGGTGAAGACGCCAGTGGA
OCT4	GTGCCGTGAAGCTGGAGAA	TGGTCGTTTGGCTGAATACCTT
SOX2	GTGAGCGCCCTGCAGTACAA	GCGAGTAGGACATGCTGTAGGTG
Nanog	CCTGTGATTTGTGGGCCTGA	CTCTGCAGAAGTGGGTTGTTTG

Supplementary Video S1: DOX•HCl uptake in mammospheres with seperated fluorescence photos and light microscope photos. See Supplementary_Video_S1

Supplementary Video S2: DOX•HCl uptake in mammospheres with merged images of fluorescence photos and light microscope photos. See Supplementary_Video_S2

Supplementary Video S3: CSOSA/DOX uptake in mammospheres with seperated fluorescence photos and light microscope photos. See Supplementary_Video_S3

Supplementary Video S4: CSOSA/DOX uptake in mammospheres with merged images of fluorescence photos and light microscope photos. See Supplementary_Video_S4
